# Treatment of Zygomatic Complex Fractures with Surgical or Nonsurgical Intervention: A Retrospective Study

**DOI:** 10.2174/1874210601812010377

**Published:** 2018-05-21

**Authors:** Thomas Starch-Jensen, Linda Busk Linnebjerg, Janek Dalsgaard Jensen

**Affiliations:** 1 Department of Oral and Maxillofacial Surgery, Aalborg University Hospital, Aalborg, Denmark; 2 Department of Otorhinolaryngology, Hospital Little Belt, Vejle, Denmark

**Keywords:** Facial injuries, Maxillofacial injuries, Open fracture reduction, Therapy, Zygomatic fractures, Surgical or nonsurgical intervention

## Abstract

**Objective:**

Evaluate the 1-year treatment outcome of zygomatic complex fractures with surgical or nonsurgical intervention.

**Materials and Methods:**

One hundred and forty-two consecutive patients with a zygomatic complex fracture were enrolled. Sixty-eight patients underwent surgical intervention and 74 patients nonsurgical intervention. The 1-year examination evaluated cosmetic and functional outcome including malar symmetry, ocular motility, occlusion, mouth opening, neurosensory disturbances, and complications.

**Results:**

Forty-six patients allocated to surgical intervention responded to the 1-year follow-up examination. Satisfying facial contour and malar alignment was observed in 45 patients. All patients presented with identical position of the eye globe without enophthalmos and normal ocular movement. A habitual occlusion was seen in all patients with a mean interincisal mouth opening without pain of 49 mm. One patient presented with minor ectropion. Wound infection occurred in five patients. Persistent infraorbital neurosensory disturbance was described by 19 patients. The 1-year radiographic examination showed adequate fracture alignment in all patients with satisfying facial contour. However, dissimilar position of the orbital floor was seen in three patients having orbital reconstruction. None of the patients were re-operated or needed secondary correction of the zygomatic complex or orbital floor.

**Conclusion:**

Surgical intervention is an effective treatment modality of depressed zygomatic complex fractures, whereas a nonsurgical approach is often used for nondisplaced fractures. Most zygomatic complex fractures can be treated solely by an intraoral approach and rigid fixation at the zygomaticomaxillary buttress. Further exposure of the zygomaticofrontal junction or inferior orbital rim is necessary for severely displaced fractures, which require additional fixation.

## 1 INTRODUCTION

The zygomatic bone defines the anterior and lateral projection of the face and articulates with the frontal, sphenoid, temporal, and maxillary bones. The zygomatic complex is responsible for the protection of the orbital contents and the mid-facial contour. Fracture of the zygomatic complex is one of the most common facial injuries in maxillofacial trauma and predominately appears in young adult males [1-5]. The etiology of zygomatic complex fractures primarily includes road traffic accidents, violent assaults, falls and sports injuries [1-5]. However, there is geographic and sociodemographic variation in the epidemiology of maxillofacial fractures due to socioeconomic, cultural and environmental factors. The main clinical features of zygomatic complex fractures include diplopia, enophthalmos, subconjunctival ecchymosis, extraocular muscle entrapment, cosmetic deformity with depression of the malar eminence, facial widening, malocclusion and neurosensory disturbances of the infraorbital nerve [6]. Diagnosis of zygomatic complex fractures is usually clinical, with confirmation by computed tomography (CT) scan [6].

The integrity of the zygomatic complex is fundamental in maintaining normal facial width and prominence of the cheek. Zygomatic complex fractures with no or minimal displacement are often treated without surgical intervention, whereas fractures with functional or esthetic impairments in the form of diplopia, extraocular muscle entrapment, malocclusion, restricted mouth opening and/or depression of the malar prominence often necessitate surgical intervention. Various surgical approaches and treatment strategies have been proposed to obtain successful treatment outcome, including the Gilles temporal approach, coronal, eyebrow, upper eyelid, transconjunctival, infraciliary lower eyelid, and intraoral vestibular approaches [4-7]. The surgical approach for adequate reduction of zygomatic complex fractures must provide maximum necessary exposure of the fractured segments, minimize the potential for injury to facial structures, and ensure a good functional and cosmetic result. The Gilles temporal approach has been a commonly used surgical technique for the reduction of zygomatic complex fractures. However, this surgical approach is associated with a facial scar in the hairline and risk of facial nerve palsy. Moreover, further exposure of the zygomaticofrontal junction or the inferior orbital rim is required for placement of mini-plates fixation in case of an unstable zygomatic complex fracture. Surgical reduction of zygomatic fractures by an intraoral surgical approach was first described in 1909 by Keen [8], and several studies have subsequently documented the treatment outcome after open reduction of zygomatic complex fractures by an intraoral surgical approach [9-17].

The objective of the present retrospective study was to assess the 1-year clinical and radiographic outcome after surgical or nonsurgical treatment of zygomatic complex fractures.

## 2 MATERIALS AND METHODS

### 2.1 Patients

One hundred and forty-two consecutive patients (113 males and 29 females) with a zygomatic complex fracture were admitted to the Department of Oral and Maxillofacial Surgery, Aalborg University Hospital, Denmark. The average age of the patients was 42.2 years (range 9-97). The mechanism of injury was accidents at work (4%), sports injuries (12%), bike accidents (15%), assaults or interpersonal violence (21%), road traffic accidents (23%), and falls (25%). The time interval between injury and the initial consultation ranged from 0 to 60 days (mean of 3 days). Six patients were in the intensive care unit at the initial consultation. The zygomatic complex fractures were confirmed by CT-scan. Twenty-nine of the patients had concomitant facial fractures involving the nose (8), mandible (10) and Le Fort I/II/III fractures (11).

### 2.2 Treatment Strategy

The zygomatic complex fractures were initially classified in nondisplaced and displaced. Sign and symptoms of the patient were evaluated as restricted mouth opening, diplopia, impaired eye vision, occlusal alteration, neurologic disturbance of infraorbital nerve, clinical and radiological asymmetry related to fracture displacement. The zygomatic complex fractures were treated by surgical intervention in 68 patients (48%) and without surgical intervention in 74 patients (52%).

Patients treated with nonsurgical intervention presented with insignificant flattening of the cheek (19%), restricted mouth opening (23%), diplopia (7%), malocclusion (7%), diminished eye vision (3%), extraocular muscle entrapment (1%), enophthalmos (1%) and neurosensory disturbances of the infraorbital nerve (36%). One patient with flattening of the cheek refused surgical intervention, due to no cosmetic complaint.

Patients treated with surgical intervention presented with flattening of the cheek (84%), restricted mouth opening (47%), diplopia (13%), malocclusion (19%), diminished eye vision (4%), extraocular muscle entrapment (6%), enophthalmos (1%) and neurosensory disturbances of the infraorbital nerve (66%).

The time interval between injury and surgical intervention ranged from 0 to 11 days (mean of 3.4 days). Open reduction without mini-plate fixation was conducted in 11 patients (16%), while plate fixation with adequate mini-plate osteosynthesis was performed in 57 patients (84%). Two-point fixation involving the zygomaticomaxillary buttress and the zygomaticofrontal junction was used in 7 patients (15%), while the zygomaticomaxillary buttress and infraorbital rim were used in 3 patients (4%). Three-point fixation involving the zygomaticomaxillary buttress, zygomaticofrontal junction and infraorbital rim was used in seven patients (10%). Orbital reconstruction using a polydioxanone foil was performed in eight patients (12%). The mean length of hospitalization after surgery was 1.6 days (range 1-5).

All surgical or nonsurgical treated patients were advised not to apply pressure on the fractured side for a period of six weeks and were followed on a weekly basis for the first four weeks postoperatively, then at three months and 1-year.

### 2.3 Description of the Surgical Intervention

**Fig. (1):**
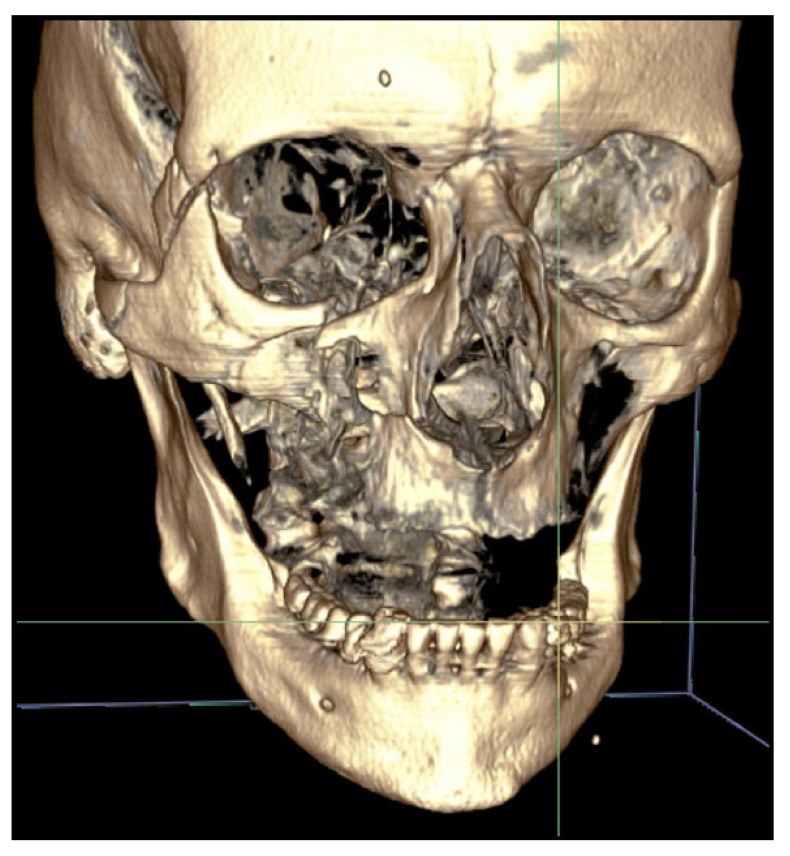
Three-dimensional CT reconstruction showing a severely dislocated zygomatic complex fracture after violent assault.

**Fig. (2):**
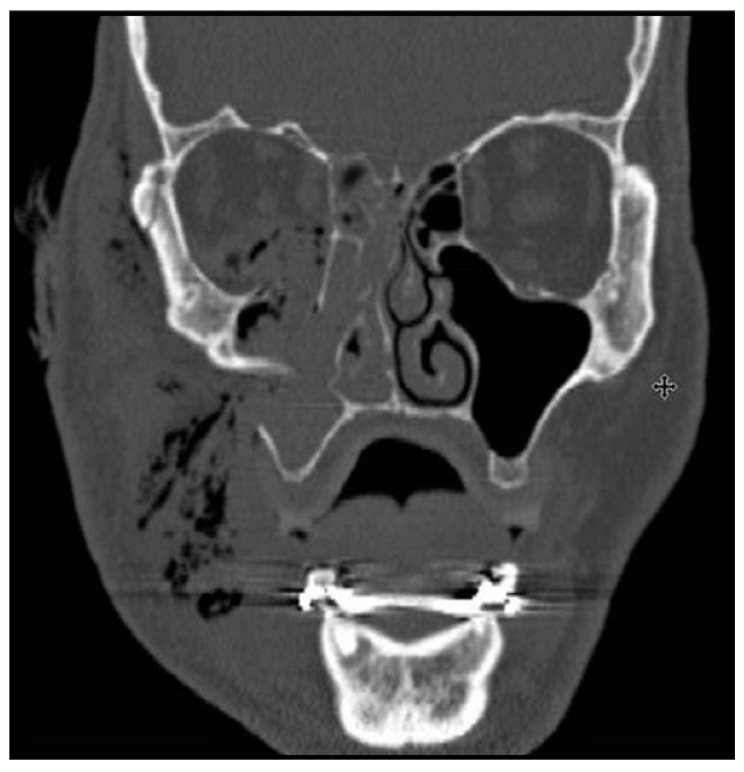
Coronal CT-scan image showing a severely dislocated zygomatic complex fracture with orbital floor fracture.

**Fig. (3):**
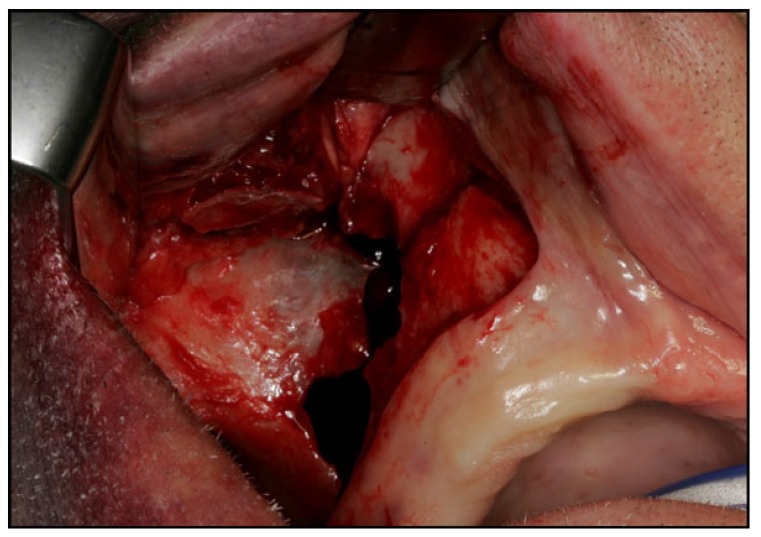
The zygomatic complex fracture was initially exposed through an intraoral vestibular approach.

**Fig. (4):**
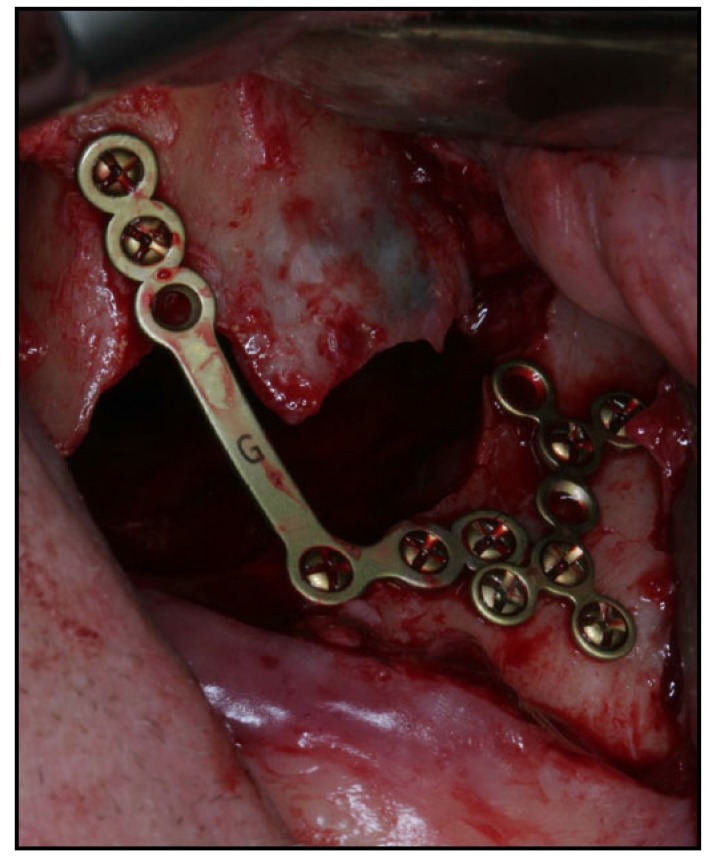
The frontozygomatic junction was exposed through a cutaneous laceration, which was extended.

**Fig. (5):**
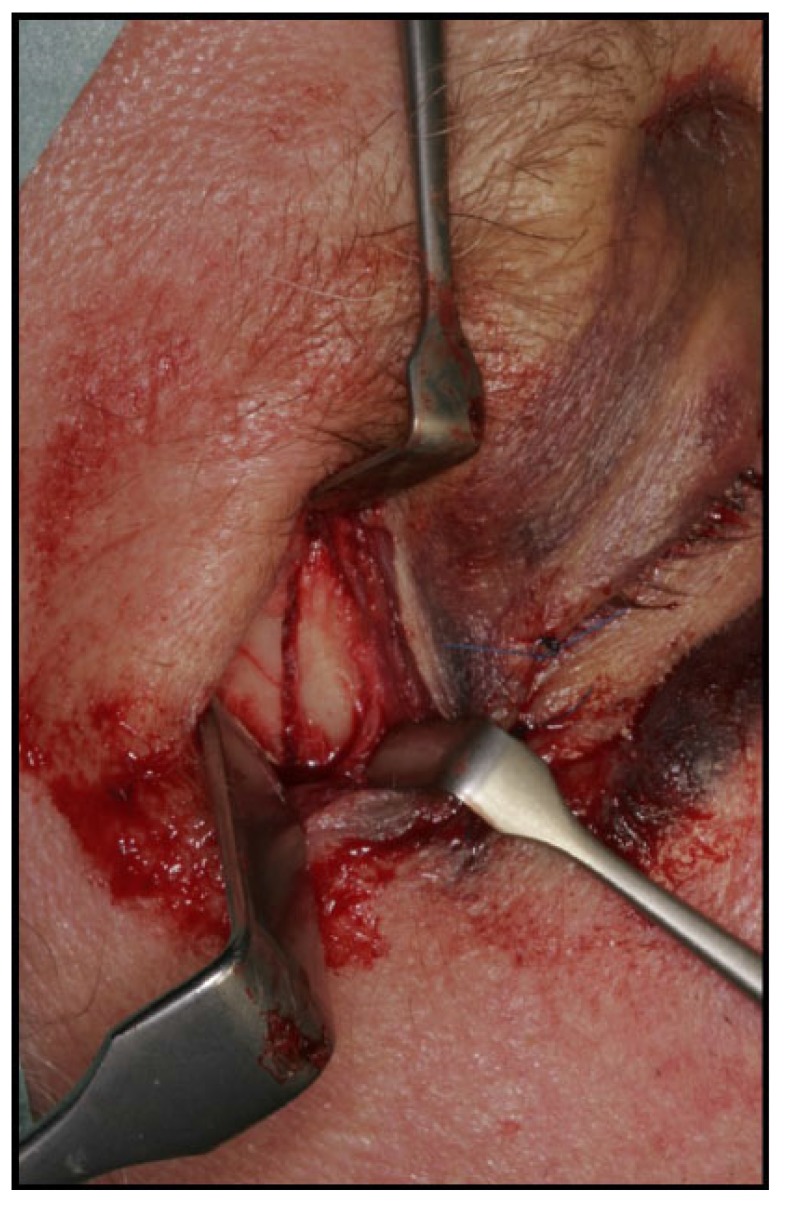
The infraorbital rim and orbital floor was exposed through a subciliary approach.

**Fig. (6):**
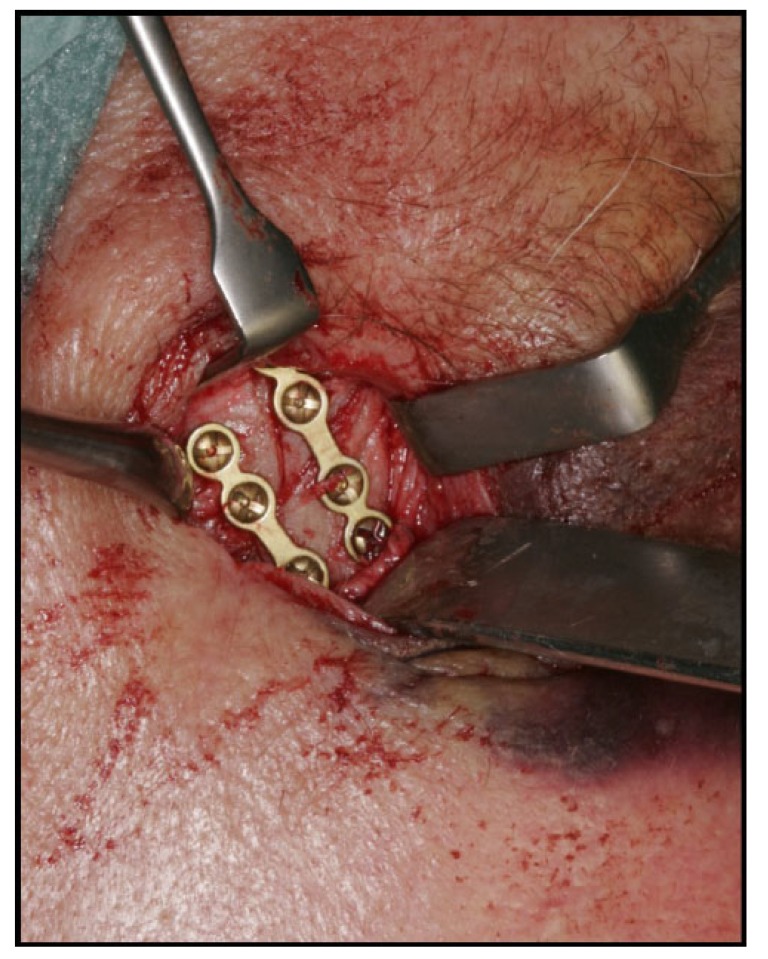
Adequate anatomic alignment and fixation with mini-plates at the zygomaticomaxillary buttress.

**Fig. (7):**
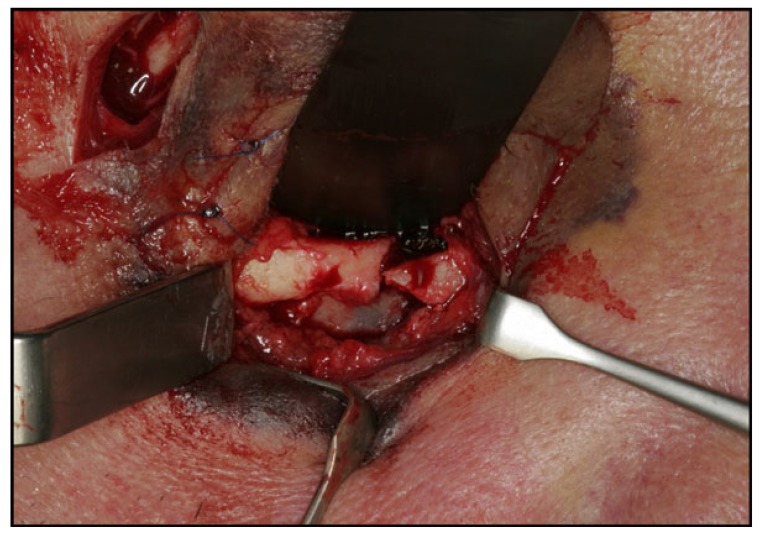
Adequate anatomic alignment and fixation with mini-plates at the zygomaticofrontal junction.

**Fig. (8):**
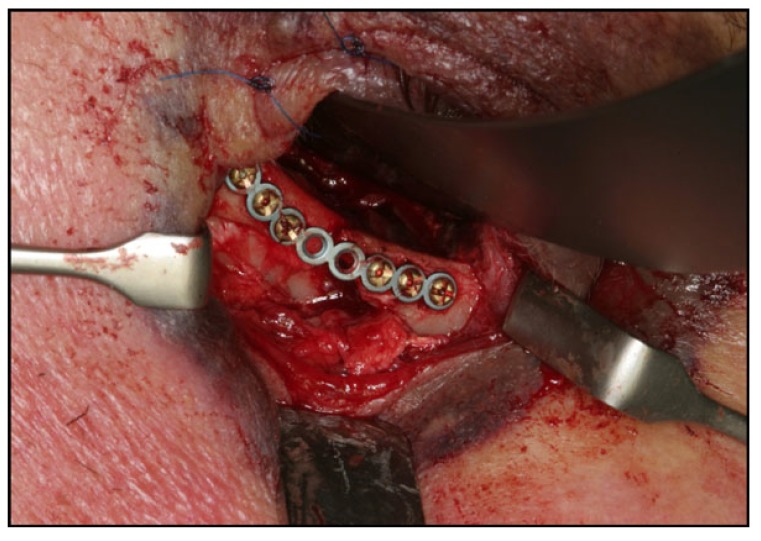
Adequate anatomic alignment and fixation with mini-plates at the infraorbital rim.

Surgical treatment of zygomatic complex fractures was performed in general anesthesia with an oral or nasotracheal intubation. The surgical intervention was conducted by different surgeons using a similar surgical technique. Initially, a forced duction test for ocular motility was conducted to determine the presence or absence of extraocular muscle entrapment. After local anesthesia, an upper buccal vestibular incision was made from the canine to the first molar. The mucoperiosteum was reflected exposing the nasomaxillary and zygomaticomaxillary buttresses. The infraorbital nerve was identified and protected. Under direct vision, the depressed zygomatic complex was elevated and manipulated into its proper anatomical alignment by Rowe's elevator, whilst the contour of the infraorbital rim and the frontozygomatic junction were palpated. Though the fracture reduction was stable and showed adequate anatomic alignment, the zygomatic complex fracture was almost always stabilized with mini-plates fixation at the zygomaticomaxillary buttress. If the fracture reduction was inadequate anatomic aligned or the zygomatic complex was considered unstable, the zygomaticofrontal junction and/or the infraorbital rim was exposed for second or third fixation points. Finally, a forced duction test for ocular motility was conducted to determine the presence or absence of extraocular muscle entrapment. The sulcus incision was closed using absorbable sutures and extraoral closure was done in layers with Vicryl 4-0 and Prolene 5-0 sutures. A patient example is illustrated (Figs. **1**-**8**).

### 2.4 The 1-year Clinical Evaluation

The 1-year clinical examination comprised an evaluation of following features: facial contour and malar alignment, eye globe position, ocular motility, diplopia, dental occlusion, interincisal mouth opening, patients’ perception of infraorbital paresthesia, pain and tenderness, postoperative complications and need for re-operation or secondary correction of the zygomatic complex. The position of the eye globe was based solely on a clinical judgment without the use of an exophthalmometer.

### 2.5 Radiographic Evaluation

The quality of fracture reduction was estimated on postoperative CT-scans. The diastasis between the fracture ends was measured. Fractures exhibiting a bone diastasis ≤3 mm were defined as adequate anatomic alignment, whereas fractures exhibiting a bone diastasis ≥3 mm were defined as inadequate anatomic alignment. Moreover, the facial contour and malar prominence was assessed as well as the position of the eye globe and the orbital floor.

The 1-year radiographic examination involved an evaluation of the facial contour and malar symmetry as well as the position of the eye globe and the level of the orbital floor. Mal-union and displacement of fixation plates and screws were also registered.

## 3 RESULTS

### 3.1 Nonsurgical Intervention

A total of 23 patients (31%) allocated to nonsurgical treatment of zygomatic complex fractures responded to the 1-year follow-up examination. Persistent minor flattening of the cheek was seen in 3 patients (13%). All patients presented with identical eye globe position without enophthalmos and normal ocular movement. A habitual dental occlusion was seen in all patients with a mean unassisted interincisal opening without pain of 49 mm (range: 39-58). No infraorbital neurosensory disturbances were described. The 1-year radiographic evaluation showed satisfying facial contour in all patients. None of the patients treated without surgical intervention needed secondary correction of the zygomatic complex or orbital floor.

### 3.2 Surgical Intervention

A total of 46 patients (68%) allocated to surgical treatment of zygomatic complex fractures responded to the 1-year follow-up examination. Satisfying facial contour and malar alignment was observed in 45 patients (98%). All patients presented with an identical position of the eye globe without enophthalmos and normal ocular movement. A minor degree of ectropion was observed in one patient. Postoperative wound infection occurred in five patients (11%). The osteosynthesis material was removed in five patients (11%) due to patient wish or wound infection. A habitual dental occlusion was seen in all patients with a mean unassisted interincisal opening without pain of 49 mm (range: 32-65). Infraorbital neurosensory disturbances were described by 19 patients (41%), which were rated as 1 on the visual analog scale by all patients. Postoperative and 1-year CT-scans showed adequate anatomic alignment in all patients with satisfying facial contour. However, asymptomatic loosening of the mini-plates was seen in one patient and dissimilar position of the orbital floor was observed in three patients having orbital reconstruction (38%). None of the patients were re-operated or needed secondary correction of the zygomatic complex or orbital floor.

## 4 DISCUSSION

The objective of the present retrospective study was to assess the 1-year clinical and radiographic outcome after treatment of zygomatic complex fractures with a surgical or nonsurgical intervention. A retrospective study design involves various methodological confounders including pre-recorded and patient-centered data, no standardized treatment strategy or randomizing between treatment modalities, and no homogenization of trauma or included patients. Hence, the conclusions drawn from the results of this retrospective study should be cautiously interpreted.

Re-establishment of the facial malar contour, position of the eye globe, dental occlusion as well as a normal mandibular range of motion is essential in the treatment of zygomatic complex fractures. The zygomatic complex consists of four pillars attached by four suture lines to the frontal, sphenoid, temporal, and maxillary bones. Thus, adequate anatomic alignment at all four suture lines is required to avoid changes in the facial appearance, position of the eye globe and functional impairment. Various surgical approaches and treatment strategies have been proposed to obtain a satisfying anatomic alignment of the zygomatic complex fracture and a successful treatment outcome [4-7]. However, no consensus agreement regarding the number of fixation points, sequences of rigid fixation or surgical approach exist.

Displaced and/or comminute zygomatic complex fractures are often treated by open reduction and internal mini-plate fixation. The fracture anatomy, functional deficit, fracture severity and the resultant esthetic determines the treatment strategy, surgical approaches and number and location of mini-plates for fixation. The intraoral surgical approach offers several advantages compared to the extraoral approach including no visible skin scar, visualization of the fracture line at the zygomaticomaxillary buttress and the infraorbital nerve, closer and more precise application of force by the operator, placement of fixation plates at the zygomaticomaxillary buttress through the same intraoral incision, and diminished morbidity. However, further exposure of the zygomaticofrontal junction or the inferior orbital rim is necessary for severely displaced zygomatic complex fractures, which require additional rigid fixation or reconstruction of the orbital floor.

In the present study, a sequential surgical treatment strategy has been used exposing the zygomaticomaxillary buttress as the first approach, followed by either the frontozygomatical junction and/or the infraorbital rim, when adequate anatomic alignment could not be achieved solely by the intraoral approach. The 1-year clinical and radiographic evaluation after open reduction of zygomatic complex fractures showed satisfying facial contour in 98% of the patients and anatomic alignment of the zygomatic complex. Minimal persistent flattering of the malar prominence was observed in one patient with a severely displace zygomatic complex fracture having three-point fixation and reconstruction of the orbital floor. All patients presented with a normal mandibular range of motion, habitual dental occlusion, normal ocular movement and identical position of the eye globe without enophthalmos. Persistent infraorbital sensory disturbances were observed in 41% and dissimilar position of the orbital floor was seen in 38% of the patients having orbital reconstruction.

Open reduction of zygomatic complex fractures solely by an intraoral surgical approach has previously been described in the literature [9-17]. Thirty patients with zygomatic complex fractures were treated with an intraoral approach and one point fixation at the zygomaticomaxillary buttress [16]. Clinical and radiographic evaluation disclosed no paresthesia, pain or cosmetic disfiguration after six months. However, comminuted zygomatic complex or orbital fractures were excluded from the study [16]. A retrospective study involving one hundred fifty-three patients with zygomatic complex fractures were analyzed after a minimum of three months revealed that the zygomaticomaxillary buttress was the most commonly used surgical approach and the infraorbital rim and/or the zygomaticofrontal junction was solely exposed in severely displaced zygomatic complex fractures. Moreover, a statistically significant difference between fracture displacement and surgical approach for the infraorbital rim and zygomaticofrontal junction was disclosed [15]. Another retrospective study involving 379 patients revealed a successful treatment outcome after zygomatic complex fracture alignment using an intraoral approach in 203 patients [11]. Additional fracture exposure was required in 124 patients involving the zygomaticofrontal junction and zygomaticomaxillary buttress, while orbital floor reconstruction and infraorbital rim fixation was necessary for 39 and 28 patients, respectively. The results of abovementioned studies seem to be in accordance with the results of the present retrospective study.

Complications following surgical treatment of zygomatic complex fractures include diplopia, enophthalmos, extraocular muscle entrapment, facial asymmetry, persistent flattening of the malar prominence, neurosensory disturbances of the infraorbital nerve, malocclusion and limited mandible range of motion [6, 7, 18]. It has been concluded that the risk of complications after zygomatic complex fractures increases with a higher level of complexity [18]. Complications of zygomatic complex fractures can occur from the initial trauma, from the surgical intervention, or from inaccurate surgical treatment. It has been reported that up to 5.5% of patients required a second procedure for zygomatic complex fractures within 4 weeks of initial repair due to inadequate reduction [19]. Persistent neurosensory disturbances due to infraorbital nerve injury after zygomatic complex fractures are a common clinical feature. In the present study, infraorbital neurosensory disturbances were described in 41% of the patients having surgical intervention, whereas none of the nonsurgically treated patients suffered from infraorbital neurosensory disturbances, after 1-year. These results are in accordance with a long-term study reporting more permanent neurosensory disturbances of the infraorbital nerve in patients with complex fractures compared to isolated orbitozygomatic fractures [20]. In the present study, 38% of the patients having orbital reconstruction presented with a dissimilar radiographic position of the orbital floor, after 1-year. However, none of the patients had an asymmetrical eye globe position, enophthalmos or diminished eye vision. Consequently, secondary correction of the zygomatic complex or orbital floor diplopia was not performed.

Posttraumatic enophthalmos is a common sequela that appears after treatment of zygomatic complex fractures due to enlargement of the orbital cavity as a result of inadequate anatomic alignment, fat atrophy or fibrosis [21, 22]. Correction of post-traumatic enophthalmos requires a secondary correction of the zygomatic complex and orbital floor involving autogenous bone graft, Medpor enophthalmic implants or titanium mesh implants to restore the preinjury anatomy of the orbital cavity. Computer-assisted surgical navigation, surgical planning software and computer-generated stereolithographic models can be valuable tools for secondary correction of the zygomatic complex or orbital floor [23-27]. Recent studies have documented that the computer-assisted navigation system allows for a more accurate implementation of preoperative plans for fracture reduction, thereby yielding substantial improvements in the postoperative bilateral symmetry of the zygomatic complex fracture [24]. Hence, combining computer-assisted surgical navigation for secondary reconstruction or treatment of severely displaced zygomatic complex fractures seem to improve the treatment outcome in selected cases.

## CONCLUSION

Surgical intervention and internal fixation is an effective treatment modality of depressed zygomatic complex fractures, whereas a nonsurgical approach is often used for nondisplaced zygomatic complex fractures. The intraoral approach offers the opportunity for direct visualization of the fracture reduction and placement of fixation plates at the zygomaticomaxillary buttress. However, further exposure of the zygomaticofrontal junction or the inferior orbital rim and orbital floor is necessary for severely displaced fractures, which require additional rigid fixation.
